# Butylated Hydroxytoluene Analogs: Synthesis and Evaluation of Their Multipotent Antioxidant Activities

**DOI:** 10.3390/molecules17077645

**Published:** 2012-06-25

**Authors:** Wageeh A. Yehye, Noorsaadah Abdul Rahman, Abeer A. Alhadi, Hamid Khaledi, Seik Weng Ng, Azhar Ariffin

**Affiliations:** 1 Department of Chemistry, Faculty of Science, University of Malaya, Kuala Lumpur 50603, Malaysia; 2 Chemistry Department, Faculty of Science, King Abdulaziz University, P.O. Box 80203, Jeddah 21589, Saudi Arabia

**Keywords:** butylated hydroxyltolouene, drug-likeness properties, DPPH, lipid peroxidation, multipotent antioxidant, rule-of-five, PASS and activity prediction, thiosemicarbazide, 1,2,4-triazole, 1,3,4-thiadiazole

## Abstract

A computer-aided predictions of antioxidant activities were performed with the Prediction Activity Spectra of Substances (PASS) program. Antioxidant activity of compounds **1**, **3**, **4** and **5** were studied using 1,1-diphenyl-2-picrylhydrazyl (DPPH) and lipid peroxidation assays to verify the predictions obtained by the PASS program. Compounds **3** and **5** showed more inhibition of DPPH stable free radical at 10^−4^ M than the well-known standard antioxidant, butylated hydroxytoluene (BHT). Compound **5** exhibited promising *in vitro* inhibition of Fe^2+^-induced lipid peroxidation of the essential egg yolk as a lipid-rich medium (83.99%, IC_50_ 16.07 ± 3.51 µM/mL) compared to α-tocopherol (α-TOH, 84.6%, IC_50_ 5.6 ± 1.09 µM/mL). The parameters for drug-likeness of these BHT analogues were also evaluated according to the Lipinski’s “rule-of-five” (RO5). All the BHT analogues were found to violate one of the Lipinski’s parameters (Log*P* > 5), even though they have been found to be soluble in protic solvents. The predictive polar surface area (PSA) and absorption percent (% ABS) data allow us to conclude that they could have a good capacity for penetrating cell membranes. Therefore, one can propose these new multipotent antioxidants (MPAOs) as potential antioxidants for tackling oxidative stress and lipid peroxidation processes.

## 1. Introduction

**Figure 1:**
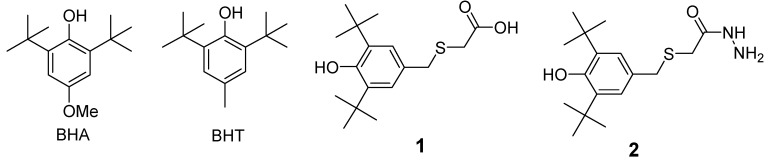
Chemical structures of compounds BHA, BHT, **1** and **2**.

Reactive oxygen species (ROS) are generally considered responsible for many cell disorders and the development of many undesired processes, including aging [1], inflammatory [2] and many others [3,4,5,6,7]. Phenolic primary antioxidants are the most active dietary antioxidants [8]. The commonly used synthetic antioxidants in foods are butylated hydroxyanisole (BHA) [9], butylated hydroxytoluene (BHT) [10] (Figure 1).

**Figure 2:**
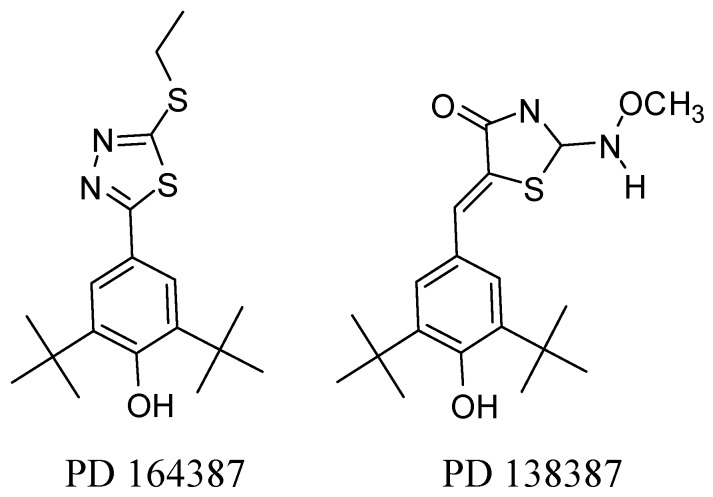
Parke-Davis COX-2 inhibitors.

BHT (CAS 128-37-0; NCI C03598) was patented in 1947 [11]. It was found that two *tert*-butyl groups flanking the OH group are required to retain *in vivo* anti-inflammatory potency [12]. Following the same route, researchers of Parke-Davis have disclosed a new class of potent, selective and orally active COX-2 inhibitors incorporating the 2,6-di-*tert*-butyl phenol moiety, such as PD 164387 and PD 138387 (Figure 2) [13,14].

Based on the Parke-Davis COX-2 inhibitors, researchers have investigated their SAR to improve COX-2 selective inhibitors containing a 2,6-di-tert-butylphenol substituent as an antioxidant moiety [15]. Their selectivity might be related to their free-radical scavenging potency [15].

On the other hand, sterically hindered phenols linked with heterocyclic rings have also been extensively studied as dual COX/5-LOX inhibitors [16,17,18]. A combination of radical scavenging properties and anti-inflammatory activity has already been proven for a number of non-steroidal anti-inflammatory drugs (NSAIDs) [19,20]. However, BHT derivatives have become attractive antioxidant or co-antioxidant groups [21]. Therefore, it is no surprise that BHT has been modified to prepare a series of new antioxidants having new medicinal properties in the pharmaceutical industry [12,22,23]. Recently, qualitative SARS and rational-design strategies for antioxidants have been used to combine multiple functions; including various multiple antioxidant properties such as radical-scavenging ability and diversified pharmacological activities to offer hybrid compounds, which can exert multiple pharmacological functions through one molecular framework [24,25,26].

Thiosemicarbazides have been reported to show antibacterial [27], antimicrobial [28,29], anti-toxoplasmagondii [29] and antioxidant [30] activities. To date acyl derivatives of thiosemicarbazide bearing BHT moiety have been rarely synthesized.

Compounds containing the 1,3,4-thiadiazole nucleus have a wide range of pharmacological activities that include antimicrobial [31], antitubercular [32], anticancer [33,34] and antioxidant [35] properties.

1,2,4-Triazoles are an important class of five membered heterocyclic compounds. 1,2,4-Triazole-5-thiones are known for their anti-inflammatory [36], selective COX-2 inhibitor [37] and antimycotic [38] and antioxidant [35] activity.

Regarding the effect of *m*-substituents, Tetsuto *et al*. [39] have evaluated the antioxidant activity of different donating substituents on a *m-*substituted phenol, and found that the *m*-substituent does not influence the antioxidant activity of a phenol at all. This is probably because a *m*-substituent shows only a small resonance effect. In contrast to this fact, previous reports found that heterocyclic systems with a halogen substituted at the *m*-position show greater antioxidant properties than those with other substituents [40,41].

Since we could not find any reports on the use of electron withdrawing groups to enhance the antioxidant activity of BHT, therefore, we used MPAO as an effective strategy to enhance the antioxidant activity of BHT even if a strong electron withdrawing group (*m*-fluoro substituent) is a basic part of the hybrid molecule.

In the present study four BHT derivatives have been synthesized. The acid-(base-)catalyzed intramolecular dehydrative cyclization reaction of acylthiosemicarbazide **3** to the corresponding 1,3,4-thiadiazole **4** and 1,2,4-triazole **5** are described. Synthesized compounds have been characterized by IR, NMR and mass spectral analysis. X-ray structures of **3** and **4** will be further discussed in this paper. Potential biological effects of new compounds were predicted based on structure-activity relationships with the PASS software. Antioxidant activities predicted by the PASS program were experimentally verified by DPPH and TBARS (thiobarbituric acid reactive substance) assays. Furthermore, a computational study for prediction of absorption, distribution (ADMET) [42] properties of the synthesized compounds was performed by determination of polar surface area (PSA), absorption (ABS) and Lipinski parameters.

## 2. Results and Discussion

### 2.1. Chemistry

Carboxylic acid **1** (Figure 1) was synthesized according to an established solvent-free procedure [43] through the reaction between 2,6-di-*tert*-butyl-phenol with formaldehyde and thioglycolic acid in the presence of di-*n*-butylamine. Hydrazide **2** (Figure 1) was synthesized using the indirect hydazination method through the reaction of 3,5-di-*t*-butyl-4-hydroxybenzyl chloride with the sodium salt of thioglycolic acid hydrazide [44].

**Scheme 1:**
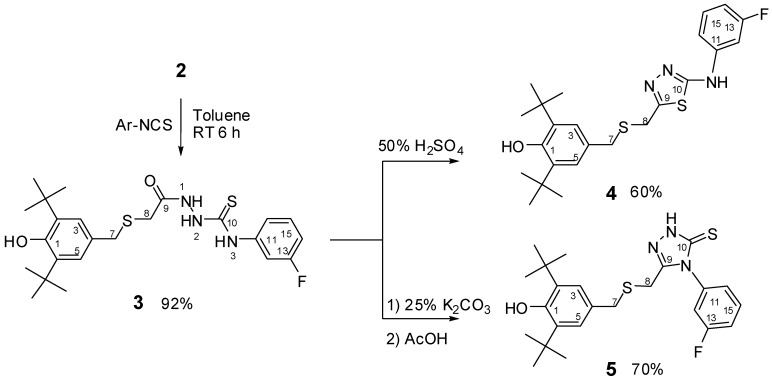
Synthesis of compounds **3**–**5**.

The synthesis of thiosemicarbazide **3**, 1,3,4-thiadiazole **4** and 1,2,4-triazole **5**, described in this study are outlined in Scheme 1. Acid hydrazide **2** was treated with 3-fluorophenylisothiocyanate to give the corresponding acylthiosemicarbazide **3** in good yield. Compound **3** under acidic and basic conditions gave thiadiazole **4** and triazole **5**, respectively. The structure of the synthesized compounds was confirmed based on their physical and spectral data. The structures of compounds **3** and **4** were further confirmed by X-ray crystallography.

The IR spectra of all synthesized compounds showed strong absorptions at 3,623–3,653 cm^−1^, attributed to free ν(Ar-O-H). Acylthiosemicarbazide **3** showed NH stretching bands between 3,285–3,340 cm^−1^, a C=O stretching band at 1,713 cm^−1^, and did not show any ν(S-H) band at 2,570 cm^−1^, while the presence of a C=S stretching band at 1,248 cm^−1^ indicated that **3** exists in the thione form in the solid-state [45,46]. Compound **4** showed two C=N stretching bands at 1,616 and 1,610 cm^−1^ attributed to the presence of the thiadiazole ring C=N. Compound **5** did not show any ν(S-H) band at 2,570 cm^−1^, while the presence of C=S stretching band at 1,252 cm^−1^ indicated that **5** exists in the thione form in the solid state [42,43].

**Figure 3:**
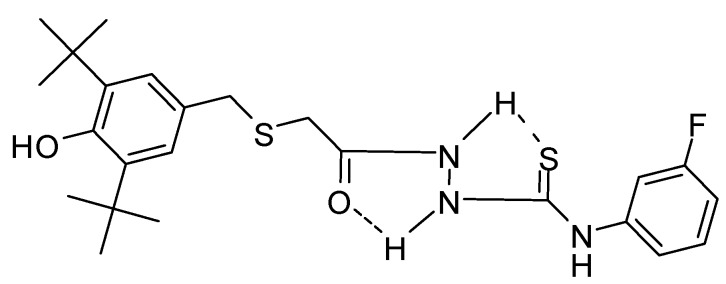
Intramolecular hydrogen bonding of thioxo form of 1-acylthiosemicarbazide **3**.

The ^1^H-NMR spectra of compound **3**, recorded in CDCl_3_, showed a singlet peak at 8.32 ppm due to N*H* attached to a phenyl group while the other two singlets at 9.26 ppm and 9.84 were attributed to the hydrazido group N*H*s. Both appear as a broad band which supports the formation of intramolecular hydrogen bonding [45,47] (Figure 3). The disappearance of the NH-1 and NH-2 groups of **3**, combined with a singlet at 11.75 ppm due to the presence of the NH-triazole of **5** suggest that a 1,2,4-triazole-5-thione, and not a 1,2,4-triazole-5-thiol was formed.

In the ^13^C-NMR spectra, the greatest differences between the acylthiosemicarbazide **3** and its cyclic derivatives **4** and **5** were found at the carbons designated C-8, C-9 and C-10. For C-8 in **3**, this carbon was bonded to C-9 (C=O). The electron-withdrawing effects of the oxygen of the C=O greatly deshielded the C-8 and caused it to appear at a high chemical shift of 33.76 ppm. After acid (base)-catalysed intramolecular dehydrative cyclizations of **3**, the carbon at the same position (C-8) in **4** and **5** were bonded to the new C-9 due to the formation of thiadiazole and thiadiazole rings, respectively, so it had a new electronic environment. This greatly lowered the chemical shift of the methylene (C-8) to values of 29.96 and 24.68 ppm in **4** and **5**, respectively. Interestingly, the heteronuclear ^13^C-^19^F coupling constants of compounds **3**, **4** and **5** were 972, 984 and 1,004 Hz, respectively.

### 2.2. Single Crystal X-ray Crystallography of Compounds ***3*** and ***4***

**Figure 4:**
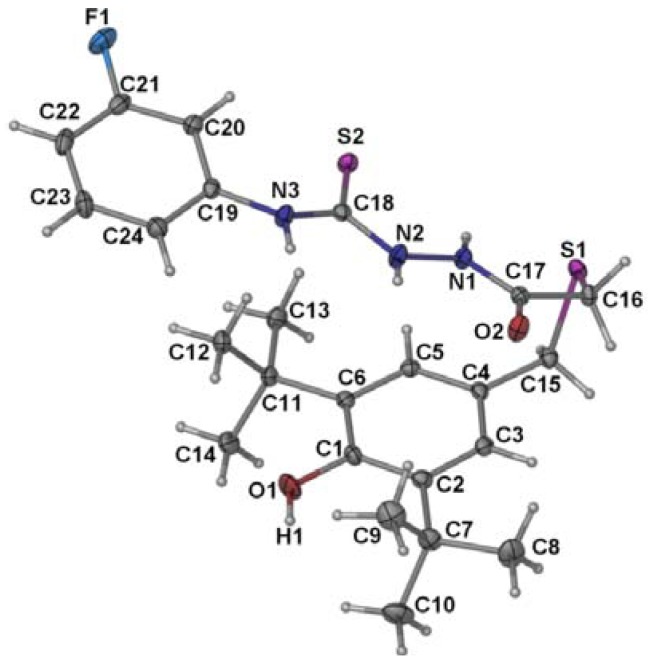
The molecular structures and labeling schemes of **3** (50% probability ellipsoids).

The crystal structure of molecule **3** is depicted in Figure 4 and the selected bond lengths and angles are given in Table 1. In the crystal the molecule exists in its thione form. The two methylene carbon atoms, C15 and C16, subtend an angle of 100.77 (7) at S1 atom. Several atom pairs of the molecule are connected via N-H…O hydrogen bonding (Table 2) in a bifurcated system to form centro-symmetric dimers. The hydroxyl group is shielded by the two di-*tert*-butyl residues and therefore is not involved in any hydrogen bonding.

**Table 1:** Selected bond lengths [Å] and bond angles [°] for **3** and **4**.

3		4	
*Bond lengths*			
S(2)-C(18)	1.6714(15)	S(2)-C(17)	1.728(6)
O(2)-C(17)	1.2417(18)	S(2)-C(18)	1.749(6)
N(1)-C(17)	1.3201(18)	O(1)-C(1)	1.376(6)
N(1)-N(2)	1.3757(17)	N(1)-C(17)	1.293(7)
N(2)-C(18)	1.3490(18)	N(1)-N(2)	1.395(6)
N(3)-C(18)	1.3563(19)	N(2)-C(18)	1.296(6)
N(3)-C(19)	1.4108(18)	N(3)-C(18)	1.365(7)
*Bond angles*			
C(16)-S(1)-C(15)	100.77(7)	C(16)-S(1)-C(15)	100.4(3)
C(18)-N(2)-N(1)	120.45(13)	C(18)-N(3)-C(19)	130.8(5)
N(2)-C(18)-S(2)	121.18(11)	C(4)-C(15)-S(1)	113.1(4)
N(3)-C(18)-S(2)	128.05(11)	C(17)-C(16)-S(1)	112.1(4)

**Table 2:** Hydrogen-bond geometry for **3** and **4**.

D-H···A	H···A [Å]	D···A [Å]	D-H···A [°]
**3**			
N(3)-H(3N)...O(2) ^#1^	2.040 (15)	2.8570 (16)	158.1 (17)
N(2)-H(2N)...O(2) ^#1^	2.019 (16)	2.7927 (17)	150.4 (17)
**4**			
N(3)-H(3N)...N(2) ^#2^	2.05 (2)	2.910 (7)	169 (5)

Symmetry transformations used to generate equivalent atoms: ^#1^ -x+1,-y+1,-z+1; ^#2^ x+1/2,-y+1/2,-z+1.

**Figure 5:**
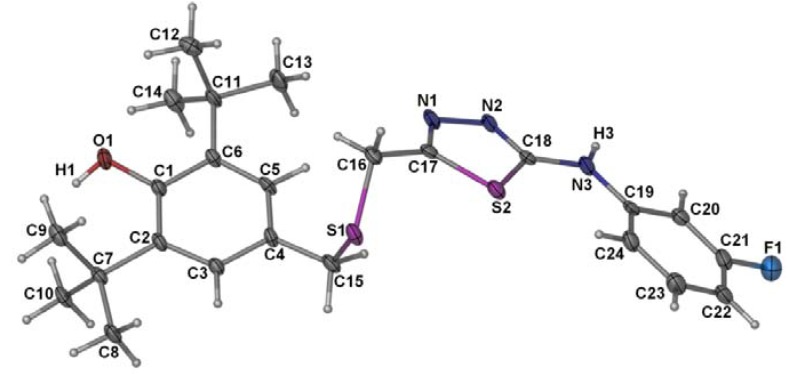
The molecular structure and labeling scheme of **4** (50% probability ellipsoids).

Figure 5 presents the molecular structure of compound **4** and Table 1 compiles the selected bond lengths and angles. The thiadiazole ring makes dihedral angles of 14.6 (3)° and 29.6(2)° with the C10-C24 and C1-C6 aromatic rings, respectively. Similar to what was observed in the structure of the semicarbazide **3**, the hydroxyl group is not involved in any hydrogen bonding as it is shielded by the two sterically hindered *tert*-butyl groups. In the crystal, pairs of N3-H…N2 hydrogen bonds (Table 2) link the molecules into centro-symmetric dimers.

The crystal data and structure refinement for compounds **3** and **4** is summarized in Table 3.

**Table 3:** Crystal data and refinement parameters for **3** and **4**.

	3	4
Empirical formula	C_24_ H_32_ F N_3_ O_2_ S_2_	C_24_ H_30_ F N_3_ O S_2_
Formula weight	477.65	459.63
Crystal system	Triclinic	Monoclinic
Space group	*P-1*	*C* 2/c
Unit cell dimensions		
*a* [Å]	9.3441 (5)	28.911 (11)
*b* [Å]	11.1548 (6)	5.731 (2)
*c* [Å]	11.7683 (6)	28.430 (11)
*α* [°]	91.189 (2)	
*β* [°]	93.388 (2)	99.330 (5)
*γ* [°]	93.224 (2)	
Volume [Å^3^]	1,222.19 (11)	4,648 (3)
Z	2	8
Independent reflections	5,587 [*R*_int_ = 0.0428]	4,202 [*R*_int_ = 0.2119]
Observed reflections [*I* > 2*σ*(*I*)]	4,988	1,848
Final *R* indices [*I* > 2*σ*(*I*)]	*R*_1_ = 0.0405, *wR_2_* = 0.1135	*R*_1_ = 0.0690, *wR_2_* = 0.1340
*R* indices (all data)	*R_1_* = 0.0448, *wR_2_* =0.1160	*R_1_* = 0.1792, *wR_2_* = 0.1820

### 2.3. Computational Evaluation of Biological Activity

The biological activity spectra of the target compounds were obtained using the Prediction of Activity Spectra for Substances (PASS) software [48]. PASS prediction tools are constructed using 20,000 principal compounds from the MDDR database (produced by Accelrys and Prous Science) [49]. The database contains over 180,000 biologically relevant compounds and is constantly updated [49]. PASS web tool has the ability to predict about 4,000 kinds of biological activity on the basis of structural formula with mean accuracy of about 90% [50,51], therefore, it is reasonable to use PASS for finding and optimizing new lead compounds. The PASS training set which has been compiled from various sources including publications, patents, chemical databases, and “gray” literatures consists of over 26,000 biological compounds and includes drugs, lead compounds, drug-candidates, and toxic substances. Algorithm of activity spectrum estimation is based on Bayesian approach. The result of prediction is presented as the list of activities with appropriate Pa and Pi ratio. Pa and Pi are the estimates of probability for the compound to be active and inactive respectively for each type of activity from the biological. It is reasonable that only those types of activities may be revealed by the compound, which Pa > Pi. If Pa > 0.3 the compound is likely to reveal this activity in experiments, but in this case the chance of being the analogue of the known pharmaceutical agents for this compound is also high. A portion of the predicted biological activity spectra (lipid peroxidase inhibitor, antioxidant, free radical scavenger and antiinflammatory) for the synthesized compounds and BHT are given in Table 4.

**Table 4:** Part of the predicted biological activity spectra for the compounds **1, 3–5** and BHT.

Mode of biological activity	1		3		4		5		BHT	
	Pa	Pi	Pa	Pi	Pa	Pi	Pa	Pi	pa	Pi
Lipid peroxidase inhibitor	0.652	0.006	0.436	0.027	0.485	0.019	0.639	0.007	0.843	0.003
Antioxidant	0.712	0.004	0.385	0.035	0.420	0.028	0.529	0.015	0.845	0.003
Free radical scavenger	0.807	0.004	0.585	0.025	0.554	0.031	0.506	0.042	0.797	0.004
Antiinflammatory	0.659	0.017	0.375	0.136	0.649	0.018	0.479	0.065	0.804	0.005

Pa—probability “to be active”; Pi—probability “to be inactive”.

### 2.4. Molecular Properties and Drug-Likeness

#### 2.4.1. Calculation of Drug-Likeness Properties

Drug-likeness can be defined as a delicate balance between various structural features, which determine whether a particular molecule is similar to recognized drugs. It generally means molecules, which contain functional groups and/or have physical properties consistent with most of the known drugs. These properties are; absorption, distribution, metabolism, and excretion in the human body like a drug. Lipinski [52] used these molecular properties in formulating his Rule of Five. The rule states that the most molecules with good membrane permeability have log*P* < 5, molecular weight <500, number of hydrogen bond acceptors <10, number of hydrogen bond donors <5. Number of rotatable bonds is important for conformational changes of molecules under study and ultimately for the binding with receptors or channels [53]. It is revealed that for passing oral bioavailability criteria, number of rotatable bond should be (≤10). The synthesized compounds **1** and **3–5** in general possessed high number of rotatable bonds (6–10) and therefore, obviously exhibit large conformational flexibility.

#### 2.4.2. Lipophilicity

Generally, lipophilic antioxidants demonstrated more potent scavenging properties than hydrophilic antioxidants. Vitamin E is a fat-soluble antioxidant [54]. Distribution of this vitamin within the membrane has been shown to alter its antioxidant potency [55]. The Partition Coefficient is a measure of how well a substance partitions between a lipid and water. It is therefore important to determine these physico-chemical properties associated with an antioxidant activity. Log*P* measurements (*P* is partition coefficient of the molecule in the water-octanol system) are a useful parameter for use in understanding the behaviour of antioxidant molecules. For calculation of log*P*, we used computed log*P* values by using ADMET predictive software as shown in Table 5. Compounds having log*P* ≥ 5 should be considered as a higher lipophilicity have higher permeation across biological membranes (but lower aqueous solubility) [56]. The log*P* values showed that the order of lipophilicity was 1-acyl-semicarbazide **3** < triazole **5** (thione form) < thiadiazole **4** and 5 (thiol form). Compounds **3–5** showed lipophilic properties with log*P* values between 6.439–6.967 while natural lipophilic antioxidant, α-tocopherol (α-TOH) (10.44) and, hydrophilic antioxidant, ascorbic acid (−1.70).

**Table 5:** Predicted ADMET and Lipinski’s parameters.

Compound	Violation of Rule of 5 (≤1)	HBA (≤10)	HBD (≤5)	Log *P* (≤5)	MW (≤500)	NROTB (≤10)	%ABS	PSA A^2^ ≤90
**1**	0	4	2	4.41	310.452	6	88.66	58.93
**3**	1	4	4	6.43	477.658	10	82.59	76.54
**4**	1	5	2	6.94	459.643	8	89.63	56.14
**5 (thione)**	1	4	2	6.84	459.643	7	92.33	48.30
**5 (thiol)**		5	2	6.96	459.643	7	92.21	48.68
**BHT**	0	1	1	4.87	220.350	2	101.81	20.81
Vitamin E *	-	-	-	10.44	430.71	-	98.73	29.74
Vitamin C *	-	-	-	−1.70	176.12	-	71.23	109.49

* Vitamin E and C are outside the “rule of 5” [52], BHT—butylated hydroxytoluene, HBA—hydrogen bond acceptor, HBD—hydrogen bond donor, NROTB—number of rotated bonds, PSA—polar surface area.

#### 2.4.3. Violations of Lipinski’s Rule of Five

It is very important to note that there are many violations of this rule among currently existing drugs and *vice versa*, but in general, an orally active drug has no more than one violation. Compounds violating more than one of the Lipinski Rules are reasonably assumed to have bioavailability problems and are therefore, presumably unsuitable as drugs [57]. However, qualifying the RO5 does not absolute guarantee that a molecule is “drug-like” [58]. Moreover, the RO5 says nothing about specific chemistry structural features found in drugs or non-drugs [59]. Polar surface area (PSA) is basically recognized as a good indicator of drug absorbance in the intestines, Caco-2 monolayers penetration, and blood-brain barrier crossing [58].

The earlier mentioned parameters were calculated for the BHT derivatives in analysis, and the results are depicted in Table 4. From the data obtained, one can notice that the synthesized compounds possess an adequate number of proton acceptor and proton donor groups to ensure efficient interaction with the hydrogen-bonding groups of the receptors. Hydrogen-bonding capacity has been also identified as an important parameter for describing drug permeability [60]. All the BHT derivatives were found to violate one of the Lipinski’s parameters (log*P* ≤ 5), although they have been found to have solubility in protic solvents. Magnitude of absorption is expressed by the percentage of absorption; Absorption percent was calculated [61] using the equation: % ABS = 109 – (0.345 × PSA). Accordingly, to their predictive low PSA (PSA values are considerably less than 90 A^2^) and high % ABS data it seems that this type of antioxidants could have a good capacity for penetrating cell membranes [53].

### 2.5. *In Vitro* Antioxidant Activities

In present study, antioxidant activities of five BHT derivatives were carried out by two well known *in vitro* antioxidant assays, DPPH and TBARS. The effects of antioxidants in the DPPH-radical-scavenging test reflects the hydrogen-donating capacity of a compound. In its radical form, DPPH • absorbs at 570 nm. The radical form of DPPH is scavenged by an antioxidant through the donation of a hydrogen atom to form a stable DPPH molecule. This leads to a colour change from purple to yellow, and a corresponding decrease in absorbance [62,63].

The thiobarbituric acid reactive substances (TBARS) assay is used for screening and monitoring lipid peroxidation. The basis of TBARS assay is the absorbance of a pink coloured complex that is monitored spectrophotometrically at 532–535 nm which is formed between thiobarbituric acid and the oxidation products of unsaturated lipids [64,65].

#### 2.5.1. *In Vitro* DPPH Free Radical Scavenging Activity

Compound **3** showed significant levels of % inhibition of DPPH radical compared to the standard antioxidant (BHT) used in the study (Table 6). As shown in this Table, thiosemicarbazide **3** exhibited strong scavengings effect on DPPH stable radical, with an IC_50_ value of 68.03 ± 1.27 µM/mL.

**Table 6:** IC_50_ value and % inhibition of the DPPH radical scavenging and lipid peroxidation inhibition assays.

Compounds	IC_50_^a^ Values (µM/mL) ± S.E.M ^b^ and Max. inhibition % ± S.E.M	
	DPPH Radical Scavenging	Lipid Peroxidation Inhibition
**1**	96.73 ± 1.87 (51.25 ± 0.82)	38.84 ± 1.54 (73.99 ± 1.30)
**3**	68.03 ± 1.27 (65.21 ± 0.55)	56.00 ± 5.05 (74.64 ± 1.68)
**4**	> 100 ^c^ (26.09 ± 0.33)	33.20 ± 2.91 (84.99 ± 1.37)
**5**	85.30 ± 1.16 (65.26 ± 0.38)	16.07 ± 3.51 (83.99 ± 1.65)
**BHT**	>100 ^c^ (25.23 ± 0.17)	36.67 ± 1.78 (79.45 ± 1.27)
**Ascorbic acid**	67.77 ± 0.17 (71.93 ± 1.61)	-
**α-TOH**	-	5.63 ± 1.09 (84.69 ± 1.23)

^a^ IC50: 50% effective concentration; ^b^ S.E.M: standard error of the mean; ^c^ did not reach 50% inhibition.

**Figure 6:**
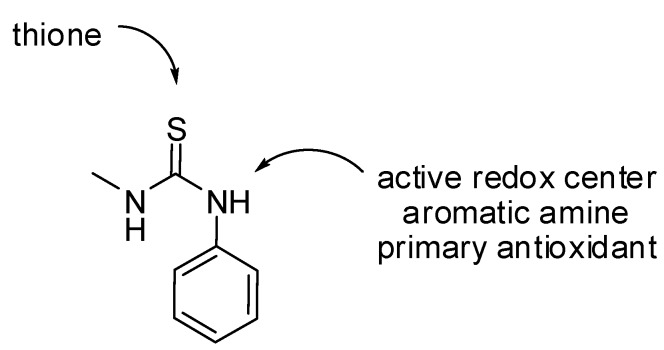
Phenylthiourea system.

This value was lower than that of the thiadiazole **4**, triazole **5**, BHT and quite close to that of ascorbic acid (67.77 ± 0.17 µM/mL), which was used as a positive control in the study, clearly indicating that thiosemicarbazide **3** has good radical scavenging activity. Presumably, significantly greater inhibition of lipid peroxidation could be attributed to the phenylthiourea system which is the active center of antioxidation, acts as the active center of containing thione and secondary aromatic amine antioxidants (Figure 6).

This result is in agreement with previous reports whereby thiourea compounds exhibited antioxidant activity, while *S*-alkylation virtually eliminated the antioxidant activity, which indicated that an unalkylated thiourea group is critical to antioxidant activity. In addition, according to the published report, thiosemicarbazide obviously showed greater antioxidant property than triazole and thiadiazole [66]. The X-ray structures (Figure 4) and also the ^1^H-NMR data of **3** revealed that the thione form is formed. Thus, the DPPH inhibition result of compound **3** suggested that the thione form is largely responsible for the antioxidant activity due to its favourable electron-donating characteristics.

In addition to that, the active redox center of compound **3** is the Ar-*N*H- (Figure 6) which increases the rate of H-atom transfer to neutralize the DPPH• by delocalization of the nitrogen electron pair over the aromatic system and the thione group, forming a stable aminyl radical, thus yielding a DPPH• inhibition of 65.21% for **3**, while ascorbic acid and BHT gave values of 71.93% and 25.23%, respectively.

Compound **4** did not show any DPPH radical-scavenging activity compared to BHT and **1**. This could be attributed to the fact that the electron-withdrawing groups produce a strengthening of the N-H bond. This behavior can be attributed to the conjugative effect of the substituents [67,68].

**Scheme 2:**
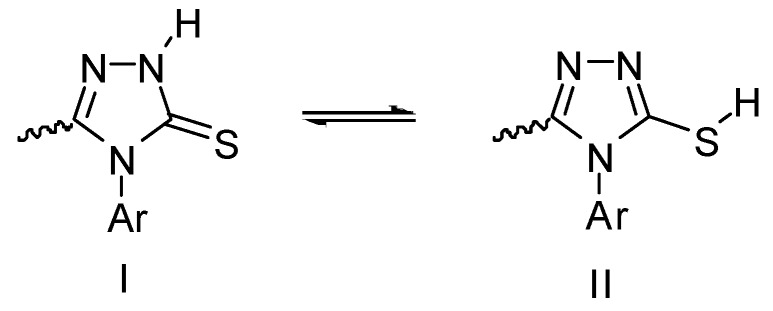
Thione I and thiol II tautomers of **5**.

Table 6 clearly shown the % inhibition of compound **5** to be higher than BHT and close to that of ascorbic acid. Presumably, like **4**, the 1,2,4-triazole is present in the thione form as a planar molecule. The increased stability of the thione form (I) over thiol form (II) (Scheme 2) has been shown theoretically, and the sulfur atom is found to be the atom with largest electron density in the molecule [69]. Thus, the chemical behaviour of the system with respect to free radicals would largely depend on the tautomeric form present in the reaction, reasonably assuming that the sulfur atom is involved in the reactive site.

Although we don’t have an exact explanation for the greater activity of **5**, we have some logical arguments. Heterocyclic systems with halogen substituents have shown greater antioxidant properties than other substituents [40,41]. Takanori *et al*. [70] studied the antioxidant activity of *o*-benzyl-substituted phenols and found that electron-withdrawing groups on benzyl substituent decreases the electron density of methylene, making hydrogen transfer to a phenoxy radical easier and regenerated the antioxidant activity of phenols. Thus, the fluorine group in compound **5** seemed to enhance free radical scavenging by making the NH of triazole ring easy to regenerate reversibly recycling antioxidant activity [71] or this might be due to its electron-donating mesomeric effect [39].

#### 2.5.2. *In Vitro* Lipid Peroxidation Activity

Contrary to the DPPH assay results, compound **3** exhibited the lowest lipid peroxidation activity yielding the highest IC_50_ value of 56 ± 5.05 µM/mL. Compound **4** was higher than BHT, with an IC_50_ value of 33.20 ± 2.91 µM/mL. Interestingly, thiadiazole **5** exhibited the strongest inhibitory activity with IC_50_ 16.07 ± 3.51 µM/mL (inhibition = 83.99%) which is two times better than the IC_50_ of the standard antioxidant BHT with IC_50_ = 36.67 ± 1.1.78 µM/mL and close to that of vitamin E (IC_50_ = 5.63 ± 1.09 µM/mL, % inhibition = 84.69). It is known that α-TOH and BHT are strongly lipophilic antioxidants due to their long hydrocarbon chain and di-tert-butyl groups similar to the tail of a fatty acid and therefore, reach a higher level of bioavailability [72]. Presumably, the significantly greater differences between the lipid peroxidation values of **3**, **4** and **5** could be attributed to the balance between the hydrophilicity of the polar moieties and the lipophilicity of the hydrocarbon moieties. Thus, the stronger inhibitory activity of compound **5** could be attributed to hydrophilic and also lipophilic effects on emulsified oils. In emulsions, which is the medium of the TBARS assay, non-polar free radical scavengers accumulate in the lipid phase and at the oil-water interface where interactions between hydroperoxides at the droplet surface and prooxidants in the aqueous phase occur [73,74,75].

Structurally, in 2010 Imtiaz *et al.* [76] found triazole derivatives to be relatively more active than the derivatives of thiadiazole. These may be regarded as substrate-like inhibitors on the basis of their structural similarity with the natural substrate of urease (*i.e*., urea). Similarly, hydroxyphenylurea derivatives exhibit 10 times higher antioxidant activity than α-TOH [24]. Thus, results of lipid peroxidation inhibition of compound **5** suggested that a 1,2,4-triazole bearing a BHT moiety and *m*-flourophenyl can donate electron/hydrogen easily. However, the *m*-flouro substituent could also enhance the antioxidant activity of BHT similar to donating groups, which could be attributed to its electron-donating mesomeric effect [41] but not to the conjugative effect [67,68], and maybe to intermolecular lipophilicity effects [77].

Log*P* calculations of **4** and **5** (Table 5) showed the thione form of **5** (log*P* = 6.84) to have only slight more polarity than **4** (log*P* = 6.94) due to thiol-thione tautomerism effect, which suggested compound **4** and **5** to have hydrophilic and lipophilic groups, which may act as amphiphilic antioxidants in one molecule rather than having to use two antioxidants separately. Thus, results of lipid peroxidation inhibition of compound **5** suggested that it to be possible candidate with promising anti-oxidant activities.

## 3. Experimental

### 3.1. General

All materials and solvents were obtained from Sigma-Aldrich. Melting points were determined on a MEL-TEMP II melting point instrument. IR spectra were recorded on a Perkin-Elmer RX1 FT-IR spectrometer. The ^1^H- (400 MHz) and ^13^C-NMR (100 MHz) spectra were recorded on JEOL ECA 400 MHz FT-NMR using CDCl_3_ or DMSO-*d*_6_ as solvent and tetramethylsilane as an internal standard. HR-mass spectra (ESI) were obtained with a MAT 95 xl-T mass spectrometer operating at 70 ev. UV-visible spectra were recorded on a UV-1650PC model UV-visible spectrophotometer.

*2-(2-(3,5-di-tert-Butyl-4-hydroxybenzylthio)acetyl)-N-(3-luorophenyl)hydrazinecarbothioamide* (**3**). To a solution of *S*-(3,5-di-*t*-butyl-4-hydroxybenzyl)thioglycolic acid hydrazide (**2**, 0.45 g, 1.39 mmol) in dry toluene or benzene (5 mL), was added 3-fluorophenylisothiocyanate (0.21 g, 1.39 mmol) and the reaction mixture was stirred for 2 h at room temperature. The resulting precipitate was collected by filtration, washed with boiled hexane, dried at room temperature, and recrystallized from toluene to give white solid, yield 92% (0.60 g), mp 180–182 °C. IR (KBr pellet), cm^−1^: ν = 3,633 (free OH, BHP), 3,340, 3,314 and 3,285 (NH), 2,873–2,961 (C-H of *t*-Bu), 1,713 (C=O), 1,248 ν (C=S). ^1^H-NMR (CDCl_3_), δ, ppm: 1.40 (s, 18H, 2 × *t*-Bu), 3.22 (s, 2H, H-8), 3.78 (s, 2H, H-7), 5.20 (s, 1H, OH, BHP), 6.62–6.66 (dt, 1H, H-14, *^3^J_F-H_* = 8.2, *^3^J* = 8, *^4^J* = 2.3 Hz), 6.85–6.87 (d, 1H, H-16, *^3^J* = 8 Hz), 7.04–7.10 (m, 1H, H-15), 7.10 (2H, H-3, H-5), 7.20–7.25 (m, 1H, H-12), 7.97 (b, 2H, NH-2, NH-3), 8.90 (1H, NH-1). ^13^C-NMR (CDCl_3_), δ, ppm: 30.30 (6C, 2 × -C(*C*H_3_)_3_, 33.51 (1C, C-8), 34.41 (2C, 2 × -*C*(CH_3_)_3_), 37.48 (1C, C-7), 106.65 (d, 1C, C-12, *^2^J_C-F_* = 104 Hz ), 110.01 (d, 1C, C-14, *^2^J_C-F_* = 88 Hz ), 114.52 (1C, C-16), 125.89 (2C, C-3, C-5), 126.93 (1C, C-4), 129.94 (d, 1C, C-15, *^3^J_C-F_* = 36 Hz), 136.41 (2C, C-2, C-6), 139.60 (d, 1C, C-11, *^3^J_C-F_* = 44 Hz), 153.35 (1C, C-1), 154.55 (1C, C-10), 163.02 (d, 1C, C-13, *^1^J_C-F_* = 972 Hz), 170.13(1C, C-9). HREIMS *m/z* 477.1918 [M]^+^ (calcd for C_24_H_32_O_2_N_3_ FS 477.1920).


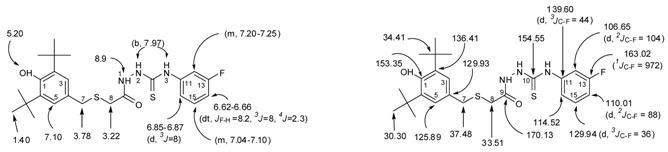


*2,6-di-tert-Butyl-4-(((5-(3-fluorophenylamino)-1,3,4-thiadiazol-2-yl)methylthio)methyl)phenol* (**4**). Thiosemicarbazide **3** (0.23 g, 0.48 mmol) was added gradually under stirring to a cooled sulphuric acid solution (50%, 5 mL) for 10 min. The reaction mixture was heated for 20 min at 100 °C. It was then poured over crushed ice under stirring. The solid precipitated was filtered, washed with water, dried and recrystallized from MeOH 4:1 H_2_O, white solid, Yield 60% (0.13 g). mp 175–177 °C. IR (KBr pellet), cm^−1^: ν = 3,623, 3,631, 3,653 (free OH, BHP), 2,873–2,958 (C-H of *t*-Bu), 1,616, 1,610 (2 C=N). ^1^H-NMR (CDCl_3_), δ, ppm: 1.40 (s,18H, 2 × *t-*Bu), 3.69 (s, 2H, H-7), 3.89 (s, 2H, H-8), 5.12 (s, 1H, OH, BHP), 6.76–6.81 (ddt, 1H, H-14, *^3^J_F-H_* = 8.5, *^3^J* = 8.4, *^4^J* = 0.8 Hz), 7.07 (2H, H-3, H-5), 7.09–7.12 (dd, 1H, H-16, *^3^J* = 8.2, *^4^J* = 0.92 Hz), 7.14–7.18 (dt, 1H, H-12, *^3^J_F-H_* = 10.4, *^4^J* = 2.7, *^4^J* = 2.4 Hz), 7.27–7.33 (dt, 1H, H-15, ^3^*J* = 8.5, *^3^J* = 8 Hz), 8–9 (b, NH). ^13^C-NMR (CDCl_3_), δ, ppm: 29.96 (1C, C-8), 30.33 (6C, 2 × -C(*C*H_3_)_3_), 34.40 (2C, 2 × -*C*(CH_3_)_3_), 36.62 (1C, C-7), 112.20 (d, 1C, C-12, *^2^J*_C-F_ = 103 Hz), 110.3 (d, 1C, C-14, *^2^J*_C-F_ = 88 Hz), 113.62 (1C, C-16_, _*^4^J_C-F_* = 11.2 Hz), 125.97 (2C, C-3, C-5), 127.50 (1C, C-4), 130.96 (d, 1C, C-15, *^3^J_C-F_* = 38.4 Hz), 136.18(2C, C-2, C-6), 141.73 (d, 1C, C-11, *^3^J_C-F_* = 42 Hz), 153.18 (1C, *C-1*), 160.17 (1C, C-9), 163.54 (d, 1C, C-13, *^1^J_C-F_* = 984 Hz), 166.59 (1C, C-10). HREIMS *m/z* 459.1759 [M]^+^ (calcd for C_24_H_30_O_1_N_3_FS_2_ 459.1814).


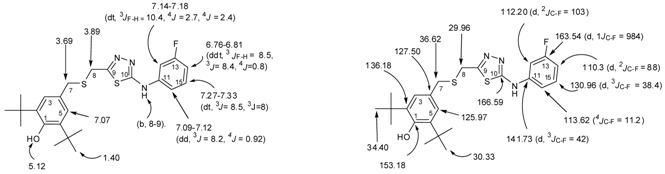


*3-((3,5-di-tert-Butyl-4-hydroxybenzylthio)methyl)-4-(3-fluorophenyl)-1H-1,2,4-triazole-5(4H)-thione* (**5**). A mixture of thiosemicarbazide **3** (0.23 g, 0.48 mmol) and potassium carbonate (25%, 5 L) was stirred for 18 h. Water (250 mL) was added and the solution was stirred for 1 h, the solution was adjusted to pH 5–6 with dilute hydrochloric acid and was kept aside for 1 h. The resulting white precipitate was filtered, washed with water, dried and and recrystallized from MeOH 4:1 H_2_O to give white sold, yield 70%, (0.16 g), mp 183–185 °C. IR (KBr pellet), cm^−1^: ν = 3,633 (free OH, BHP), 3,088, 3,014 (NH), 2,874, 2,930, 2,956 (C-H of *t*-Bu), 1,608 (C=N). 1,252.9 (C=S). ^1^H-NMR (CDCl_3_), δ, ppm: 1.41 (s, 18H, 2 × *t*-Bu), 3.36 (s, 2H, H-8), 3.64 (s, 2H, H-7), 5.18 (s, 1H, OH, BHP), 7.08 (2H, *H-3*, *H-5*), 7.14–7.19 (m, 2H, H-12, H-16), 7.21–7.26 (dt, 1H, H-14, *^3^J_F-H_* = 10.4, ^3^*J* = 8, *^4^J* = 4 Hz), 7.49–7.54 (dt, 1H, H-15, *^3^J* = 8, ^3^*J* = 8 Hz), 11.75 (b, NH). ^13^C-NMR (CDCl_3_), δ, ppm: 24.67 (1C, C-8), 30.35 (6C, 2 × -C(*C*H_3_)_3_), 34.43 (2C, 2 × -*C*(CH_3_)_3_), 36.26 (1C, C-7), 116.29 (d,1C, C-12, *^2^J*_C-F_ = 96 Hz), 117.65 (d, 1C, C-14, *^2^J*_C-F_ = 84 Hz), 124.25 (d, 1C, C-16, *^4^J_C-F_* = 16 Hz), 126.06 (2C, C-3, C-5), 126.93 (1C, C-4), 131.16 (d, 1C, C-15, *^3^J_C-F_* = 36 Hz), 134.13 (d, 1C, C-11, *^3^J_C-F_* = 40 Hz), 136.19 (2C, C-2, C-6), 150.12 (1C, C-9), 153.25 (1C, *C-1*), 162.88 (1C, C-13, *^1^J_C-F_* = 1004 Hz), 169.14 (1C, C-10). HREIMS *m/z* 459.1793 [M]+ (calcd for C_24_H_30_O_1_N_3_ F_1_^32^S_2_ 459.1814).


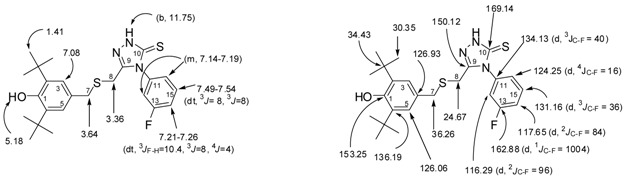


### 3.2. X-ray Crystallography

Diffraction data were measured using a Bruker SMART Apex II CCD area-detector diffractometer (graphite-monochromated Mo K radiation, =0.71073 Å). The orientation matrix, unit cell refinement and data reduction were all handled by the Apex2 software (SAINT integration, SADABS absorption correction) [78]. The structures were solved using direct method in the program SHELXS-97 [79] and were refined by the full matrix least-squares method on *F2* with SHELXL-97. Drawings of the molecules were produced with XSEED [80]. Crystal data and refinement are summarized in Table 3.

CCDC 874287 and CCDC 874288 contain the supplementary crystallographic data for **3** and **4**, respectively. These data can be obtained free of charge via http://www.ccdc.cam.ac.uk/conts/retrieving.html, or from the Cambridge Crystallographic Data Centre, 12 Union Road, Cambridge CB2 1EZ, UK; fax: (+44)-1223-336-033; or e-mail: deposit@ccdc.cam.ac.uk.

### 3.3. Antioxidant Assays

#### 3.3.1. DPPH Free Radical Scavenging Assay

DPPH radical scavenging assay was carried out according to the literature [81] with some modifications. To a range of various concentrations 100, 10, 1, 0.1 and 0.01 µM/mL of samples DPPH solution (1.0 mL, 200 µM in DMSO) was added. 22.03–47.76 mg (1 × 10^−4^ M) of test compound was dissolved in DMSO (1.0 mL, 100%) as a stock solution. This stock solution was then diluted to a range of final extraction concentrations 100, 10, 1, 0.1 and 0.01 µM. A negative control with the same DPPH concentration in DMSO without sample was used. Each assay was carried out in triplicates. The mixture was then incubated in dark for 60 min at room temperature. Absorbance at 570 nm for each sample was then measured. Ascorbic acid was used as positive control. The free radical scavenging activity of the compounds was calculated as a percentage of radical inhibition by using the formula:


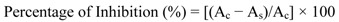


in which A_s_ = Absorbance of the compounds/ positive control and A_c_ = Absorbance of control (DPPH solution and DMSO). To determine the concentration required to achieve 50% inhibition (IC_50_) of DPPH radical, the percentage of DPPH inhibition for each compound was plotted against extract concentration.

#### 3.3.2. Lipid Peroxidation Inhibition Assay

The lipid peroxidation inhibition assay was carried out according to the reported method with some modifications [82]. Fowl egg yolk, comprising mainly of phospholipids, proteins and triacyglycerol, was used as an alternative to rat liver microsomes and linoleic acid. The reactive mixture for the induction of lipid peroxidation included egg yolk (1.0 mL) emulsified with phosphate buffer saline (0.1 M, pH7.4), to a final concentration of 12.5 g/L and 3,000 µM FeSO_4_ (200 µL). Test compound (22.03–47.76 mg, 1 × 10^−4^ M) was dissolved in DMSO (1.0 mL, 100%) as a stock solution. This stock solution was then diluted to a range of final extraction concentrations 100, 10, 1, 0.1, 0.01 and 0.001 µM. Each assay was carried out in triplicates. The mixture was incubated at 37 °C for 1 h, after which it was treated with freshly prepared trichloracetic acid (TCA, 15%, 0.5 mL) and thiobarbituric acid (TBA, 1%, 1.0 mL). The reaction mixtures were then incubated in boiling water for 10 min. Upon cooling, the mixtures were centrifuged at 3,500 rpm for 10 min. The formation of TBARS was measured by removing 100 µL of supernatant and measuring the absorbance at 532 nm. α-TOH was used as positive control. The percentage of inhibition was calculated from the following equation:


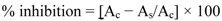


where A_s_ = Absorbance of the compound and A_c_ = Absorbance of control.

To determine the concentration required to achieve 50% inhibition (IC_50_) of phospholipid oxidation in egg yolk, the percentage of lipid peroxidation inhibition was plotted against extract concentration.

## 4. Conclusions

Our design strategy involved combining the good features of two or more antioxidants into one structure. This strategy was applied in an attempt to significantly improve the antioxidant activities of the well-known antioxidant BHT to create novel MPAOs bearing *meta* electron withdrawing groups. We have demonstrated the improvement in the free-radical scavenging capacity of BHT as determined by inhibition (25.23%) of DPPH free radical to be more than two-fold. Compound **5** is the most potent compound in the *in vitro* lipid peroxidation and obviously exhibits promising *in vitro* inhibition of Fe^2+^-induced lipid peroxidation of essential oils. The synthesized compounds **3–5** satisfied Lipinski’s RO5 and ADMET properties. RO5 and ADMET predictions can be important initial steps toward the development of novel pharmaceuticals in the fight against free radicals. Compounds **4** and **5** have exactly same molecular weight but different polarity and antioxidant activity, and therefore, PASS and MPAO design strategies can be effectively used for finding of compounds with required properties and without undesirable side effects. *m*-Flouro substituents could significantly enhance the antioxidant activity of BHT derivatives like other electron donating groups.
